# Correction: Performance of ferrite nanoparticles in inductive heating swing adsorption (IHSA): how tailoring material properties can circumvent the design limitations of a system

**DOI:** 10.1039/d4mh90095b

**Published:** 2024-09-05

**Authors:** Maxim De Belder, Alysson F. Morais, Natan De Vos, Sergey Basov, Rikkie Joris, Margriet J. Van Bael, Luc Van Meervelt, Joeri F. M. Denayer, Johan A. Martens, Eric Breynaert

**Affiliations:** a Center for Surface Chemistry and Catalysis – Characterization and Application Team (COK-KAT), KU Leuven 3001 Leuven Belgium eric.breynaert@kuleuven.be; b Quantum Solid State Physics, Department of Physics and Astronomy, KU Leuven Celestijnenlaan 200D 3001 Leuven Belgium; c Department of Chemistry, KU Leuven 3001 Leuven Belgium; d Department of Chemical Engineering, Vrije Universiteit Brussel 1050 Brussels Belgium; e NMRCoRe – NMR – X-Ray platform for Convergence Research, KU Leuven 3001 Leuven Belgium

## Abstract

Correction for ‘Performance of ferrite nanoparticles in inductive heating swing adsorption (IHSA): how tailoring material properties can circumvent the design limitations of a system’ by Maxim De Belder *et al.*, *Mater. Horiz.*, 2024, **11**, 4144–4149, https://doi.org/10.1039/d4mh00377b.

The authors regret the omission of Sergey Basov, Rikkie Joris and Margriet J. Van Bael (Quantum Solid State Physics, Department of Physics and Astronomy, KU Leuven, Celestijnenlaan 200D, 3001 Leuven, Belgium) from the author list of the original published article. The corrected author list and affiliations are as shown in this notice.

The following text should be added to the “Author contributions” section: M. J. Van Bael contributed the resources enabling the SQUID magnetisation measurements and supervised these experiments. S. Basov and R. Joris: SQUID magnetisation measurements.

In addition, the following text should be added to the “Acknowledgements” section: SB, RJ and MJVB acknowledge funding from the KU Leuven C1 program (grant no. C14/18/074) and from the FWO under the Excellence of Science (EOS) program (grant no. G0J4922N).

The Royal Society of Chemistry apologises for these errors and any consequent inconvenience to authors and readers.

